# Baicalin suppresses autophagy-dependent ferroptosis in early brain injury after subarachnoid hemorrhage

**DOI:** 10.1080/21655979.2021.1975999

**Published:** 2021-10-27

**Authors:** Bao Zheng, Xiwei Zhou, Lujun Pang, Yanjun Che, Xin Qi

**Affiliations:** Department of Neurosurgery, Jingjiang People’s Hospital, Jingjiang, Jiangsu, China

**Keywords:** Baicalin, ferroptosis, brain injury, SAH

## Abstract

Early brain injury, characterized by massive cell apoptosis or death, is identified as a critical pathophysiological process during subarachnoid hemorrhage (SAH). Ferroptosis, a class of autophagy-dependent cell death discovered in 2012, is induced by iron-dependent lipid peroxidation accumulation. The present study was designed to study the role of baicalin in autophagy-dependent ferroptosis in early brain injury after SAH. Neurological scores and brain water content were measured to evaluate brain injury. Measurement of iron ion, malondialdehyde (MDA), lipid reactive oxygen species was conducted for ferroptosis evaluation. Immunofluorescence staining, western blotting, and flow cytometry analysis were used to evaluate autophagy and apoptosis. First, we observed that, compared with sham rats, SAH rats had lower neurobehavioral scores. Next, baicalin was proven to decrease the Fe^2+^, malondialdehyde, and ROS levels in the brain tissues of rats. Also, baicalin was confirmed to suppress the beclin1, LC3-II, and LC3-I protein levels in rat brain tissues. Moreover, we found that baicalin inhibited neuronal apoptosis. Finally, the effects of baicalin on brain injury in the SAH rats were verified. Overall, our results demonstrated that baicalin suppressed autophagy-dependent ferroptosis in EBI after SAH.

## 1. Introduction

Subarachnoid hemorrhage (SAH), mainly inducing by a ruptured aneurysm, has been identified as a category of hemorrhage stroke with high morbidity and mortality globally [1–4]. Neurodegeneration induced by the ciliospinal sympathetic center ischemia may be responsible for permanent miosis following SAH [5]. Previous studies have mainly focused on delayed cerebral ischemia or cerebral vasospasm following SAH [6–9]. Nonetheless, in recent years, research has demonstrated that the commonest risk factor for disability and death in aneurysmal SAH patients is early brain injury (EBI) [10,11]. EBI leads to the poor prognosis of SAH [10]. In EBI, multiple forms of cell death have been identified and elucidated, including ferroptosis [12,13]. Unlike apoptosis or necrosis, ferroptosis refers to the autophagy-dependent cell death [14], which is known as a non-apoptotic pattern of regulated cell death [15,16]. Previous research has confirmed that targeting ferroptosis ameliorates EBI after SAH [17,18]. However, studies on autophagy-dependent ferroptosis in EBI after SAH are still limited, and an improved understanding of the specific regulatory mechanism of autophagy-dependent ferroptosis in EBI after SAH is necessary.

Baicalin, a flavonoid compound, can be extracted from the *Scutellaria baicalensis Georgi* (a traditional Chinese medicinal herb). Previous studies identified that baicalin reduces blood–brain barrier permeability and alleviates brain damage in focal cerebral ischemia because of its anti-oxidative property [19–21]. Baicalin can be quickly absorbed and stably exert its effects for more than 12 hours in the plasma, exhibiting as a multi-therapeutic agent [22,23]. Over the past years, the role of baicalin has been widely studied. Previous research has demonstrated that baicalin exerts a protective role in various diseases [22–24]. For example, baicalin inhibits the fibrogenic process of human renal proximal tubular cells in diabetic milieu [25]. Baicalin inhibits lipopolysaccharide-induced inflammatory response by regulating miR-21 in H9c2 myocardial fibroblasts [26]. Baicalin was also reported to attenuate chronic hypoxia-evoked pulmonary hypertension by targeting adenosine A receptor-evoked Protein Kinase B signaling [27]. Importantly, baicalin has been reported to attenuate SAH-induced brain injury [28]. Baicalin represses ferroptosis in intracerebral hemorrhage [29]. Also, baicalin reduces brain injury in SAH rats [30].

The present study was conducted to validate the protective effects of baicalin in SAH and the involvement of ferroptosis in SAH. We made the conjecture that baicalin suppressed ferroptosis and other cell death forms in EBI after SAH. In the first place, we detected the beam balance scores, neurological scores, brain water content, and the grade of SAH after SAH in the in vivo model. Next, the effects of baicalin on Fe^2+^, malondialdehyde, and reactive oxygen species levels in the brain tissues of rats were examined. Next, the effects of baicalin on beclin1 and microtubule associated protein 1 light chain 3 (LC3) protein levels in the rat brain tissues were evaluated. Subsequently, the effects of baicalin on the apoptosis of neurons were detected. Finally, whether the administration of baicalin affected the beam balance scores, neurological scores, and brain water content of the rats were assessed. The findings from our study extend the knowledge of the application of baicalin in EBI after SAH in terms of its properties to suppress autophagy-dependent ferroptosis.

## 2. Materials and methods

### 2.1. Animals

Fifty adult Sprague–Dawley rats (male, 250–280 g) purchased from Vital River (Beijing, China) were used in our study. All study protocols were authorized by the Institutional Animal Care and Use Committee of Jingjiang People’s Hospital (Jiangsu, China). All animal management was conducted in accordance with the National Institutes of Health published Guide for the Care and Use of Laboratory Animals (7th Edition). Rats were housed in cages with *ad libitum* feeding at controlled room temperature (22 ± 2°C) and humidity (60–80%) under a 12 h/12 h light/dark cycle.

### 2.2. SAH model

The establishment of the SAH model was operated based on previous studies with modifications [18,28]. In brief, rats were injected with 4 mL/kg of chloral hydrate for anesthesia and then put on a stereotactic apparatus. Subsequently, the needle was tilted at 55° in the sagittal plane and fixed anterior to the bregma (7.5 mm). The needle tip was toward the right and lowered the anterior to the chiasma (2 mm). Finally, the nonheparinized autologous femoral arterial blood (0.3 mL) was injected into a prechiasmatic cistern using a syringe pump. Rat temperature was maintained at 37 ± 0.5°C during the surgery. The rats in the sham group were injected with the same dose of saline into a prechiasmatic cistern. At last, rats were monitored for recovery and then returned to cages.

### 2.3. Animal grouping and drug administration

**Figure 1.**
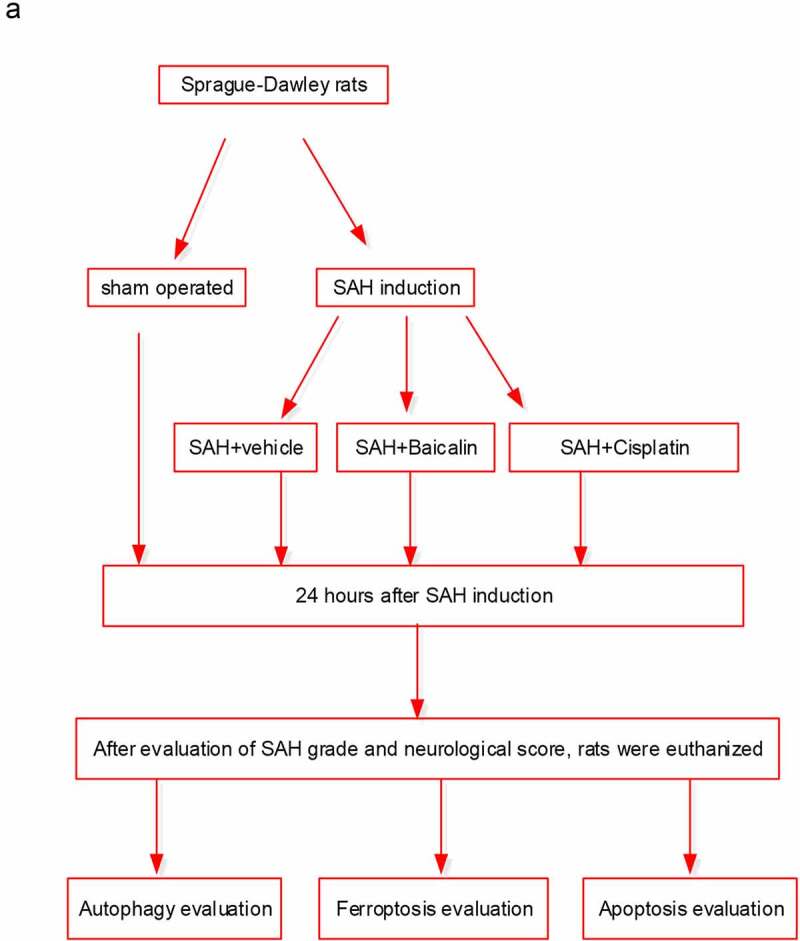
The schematic design of the animal experiments

Baicalin (Sigma, purity > 95%) was dissolved in normal saline and intraperitoneally injected into rats at 2 and 12 h after SAH operation. Cisplatin (an inhibitor of both ferroptosis and autophagy [31]) was intracerebroventricularly injected into rats 30 min before SAH operation. The rats were divided into five groups randomly: (i) the sham group, rats underwent sham operation; (ii) the SAH group, rats underwent SAH; (iii) the SAH + vehicle group, rats underwent SAH and injection of vehicle (4 mL/kg); (iv) the SAH + Baicalin group, rats underwent SAH and injection of baicalin solution (100 mg/kg, 4 mL/kg); (v) the SAH + Baicalin + Cisplatin group, rats underwent SAH, injection of baicalin (4 mL/kg), and cisplatin (14 mg/kg). All the rats were euthanized by cervical dislocation after anesthesia after SAH operation for 24 h, and brain was isolated for further exploration after behavioral tests. A schematic diagram of the animal experiments is provided in Figure 1.

### 2.4. Cell isolation and culture

Rat primary neurons were isolated from embryonic rats and cultured in the neuron culture medium with 10% fetal bovine serum (FBS; Invitrogen, USA), 1% penicillin, and streptomycin in a 5% CO_2_ incubator at 37°C as described previously [32].

### 2.5. *Establishment of an* in vitro *model of SAH*

The hemoglobin (Hb; 100 µM) solution was prepared by adding 6.67 mg of Hb powder (MERCK) into 1 mL of primary neuronal complete medium. Cells were cultured in 1.7 mL of complete medium containing 300 µL of Hb solution for 48 h.

### 2.6. Western blot

Cells were lysed by radioimmunoprecipitation assay lysis buffer. Protein samples were subjected to sodium dodecyl sulfate polyacrylamide gel electrophoresis, transferred to polyvinylidene fluoride membranes, and blocked by 5% nonfat milk powder for 1 h. Next, the membranes were incubated with primary antibodies overnight at 4°C. Primary antibodies include antibodies against Beclin-1 (ab210498, Abcam), LC3 (ab192890, Abcam), glutathione peroxidase 4 (GPX4; ab125066, Abcam) and glyceraldehyde 3-phosphate dehydrogenase (GAPDH; ab8245, Abcam). Membranes were incubated with the secondary antibodies at 37°C for 1 h, and the protein bands were visualized by an ECL Plus reagent (Applygen Technologies Inc).

### 2.7. Measurement of SAH grade

The SAH grade in filament perforation SAH was conducted following the 18-point-score method as previously described [33]. Briefly, the basal cistern was divided into six sections, and the subarachnoid blood clots in each section are evaluated. The score ranges from 0 to 3.

### 2.8. Neurological scoring

After SAH for 24 h, the neurological score was measured using the Beam balance and modified Garcia’s methods. Beam balance score was conducted to assess athletic ability [34]. The score of the modified Garcia test has six tests that evaluate spontaneous activity, symmetry in the movement of four limbs, climbing ability, response to vibrissae stimulation, forepaw outstretching, and body proprioception. The neurological core ranges from 3 to 18.

### 2.9. Iron measurement

The concentration of Fe^2+^ in the cortex and cells was detected using an iron assay kit (ab83366; Abcam). The absorbance of the cell was evaluated using a spectrophotometer (Thermo Fisher) at 593 nm.

### 2.10. MDA measurement

The concentration of MDA was detected using a lipid peroxidation MDA assay kit (ab118970; Abcam). A spectrophotometer was used to detect absorbance at 532 nm.

### 2.11. Lipid ROS measurement

The concentration of ROS was detected employing a reactive oxygen species assay kit. A spectrophotometer (Thermo Fisher) was used to detect absorbance at 525 nm.

### 2.12. Reduced glutathione (GSH) measurement

The concentration of GSH was detected using a Glutathione assay kit (CS0260; Sigma). The concentration of GSH in cells or samples was evaluated by a spectrophotometer at 412 nm.

### 2.13. Brain water content measurement

The brain weight of rats was detected with the wet/dry method, as described earlier [35]. The brain water content was calculated as [(wet weight – dry weight)/wet weight] × 100%.

### 2.14. Immunofluorescence staining of cortex tissues and primary neurons

The brain cortex tissue sections (7 mm anterior to the bregma) were incubated with anti-LC3 antibody (ab192890, 1:200; Abcam) at 4°C overnight. After being washed with PBS, the fluorescence-conjugated secondary antibody goat anti-rabbit IgG was added for incubation for 10 min at room temperature. Next, a DAPI solution was used for cell nucleus (stained blue) staining for 5 min at room temperature. Subsequently, the sections were sealed with anti-fluorescence quenching mounting solution after drying. LC3 was stained green. Finally, the brain sections were observed and imaged. The primary neurons were fixed with 4% paraformaldehyde, permeabilized with 0.1% Triton™ X-100, and blocked with 2% BSA. Next, cells were labeled with antibody against complex V subunit d (1:250, #459,000, Invitrogen) overnight at 4°C and subsequently labeled with IgG secondary antibody conjugated with Alexa Fluor Plus 488 (green; 1:2000; A32723, Invitrogen) for 45 minutes at room temperature. Nuclei were stained with DAPI (blue). The composite image showing mitochondrial pattern of ATP5H and were captured using the scale bar of 20 μm.

### 2.15. Terminal deoxynucleotidyl transferase-mediated dUTP nick-end labeling (TUNEL) staining

TUNEL staining was performed using an In Situ Cell Death Detection Kit (Roche, Germany) according to the manufacturer’s instructions. Briefly, the neurons were incubated with the kit at 37°C for 3 h. TUNEL cells were stained green. A DAPI solution was used for the cell nucleus (stained blue) staining for 5 min at room temperature. At last, a fluorescence microscope was used to observe TUNEL positive cells.

### 2.16. Flow cytometry analysis

Primary neurons (5 × 10^6^) were collected and digested by trypsin (1 mL) without ethylenediamine tetraacetic acid for 1 min. After centrifugation at 1,000 × g for 3 min, the supernatant was removed. After washing twice with pre-cooled PBS, cells were resuspended in 1X Annexin V binding buffer. Cells were treated with Annexin V-fluorescein isothiocyanate (FITC; 1.25 µL) and propidium iodide (10 µL) for 15 min in the darkness using an Annexin V-FITC cell apoptosis detection kit (Beyotime). Apoptosis was measured by FACSCalibur™ flow cytometry.

### 2.17. Data statistics

All data are presented as the means ± standard deviation. Data analysis was performed using SPSS 23.0 (IBM SPSS, Chicago, IL, USA). Statistical significance among three or more groups was analyzed by one-way ANOVA followed by Tukey’s *post-hoc* test, while that between two groups was calculated using Student’s t-test. P < 0.05 was regarded to have statistical significance.

## 3. Results

### 3.1. The neurobehavioral scores and SAH grading of the SAH rats

**Figure 2.**
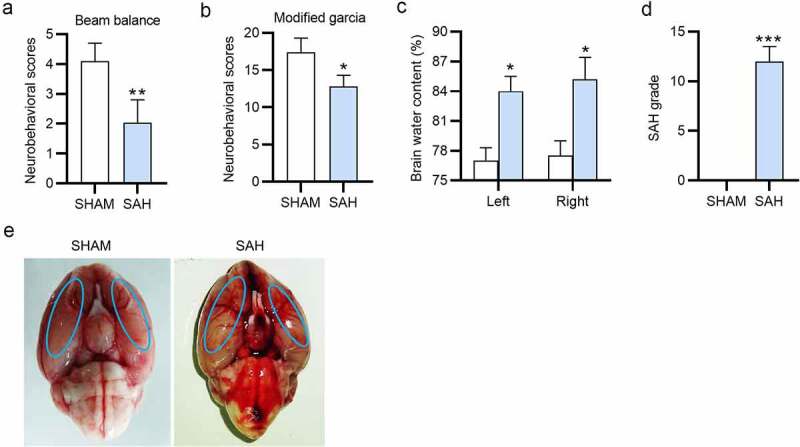
**The neurobehavioral scores and SAH grading of the SAH rats**. (a) The beam balance scores of the sham and SAH rats. (b) The neurobehavioral scores of the sham and SAH rats. (c) The brain water content (%) of the sham and SAH rats. (d) The SAH grade of the sham and SAH rats. (e) The macroscopic pictures of the brains isolated from sham and rats after surgery for 24 h. There are 10 rats in each group. *P < 0.05, **P < 0.01, ***P < 0.001

To explore the role of baicalin in autophagy-dependent ferroptosis in EBI after SAH, we first established a SAH rat model. The beam balance and modified Garcia scores of the SAH rats were lower than the sham rats (Figure 2(a,b)). Moreover, the brain water content of left and right cerebral hemispheres was increased by SAH treatment (Figure 2(c)). In addition, compared to the sham group, rats in the SAH group had significantly higher SAH grade (Figure 2(d)), implying that the *in vivo* SAH rat model was successfully constructed. The representative macroscopic pictures of the rat brains in the sham and SAH groups were represented in Figure 2(e).

### 3.2. Effects of baicalin on Fe^2+^, MDA, ROS, and GSH levels

**Figure 3.**
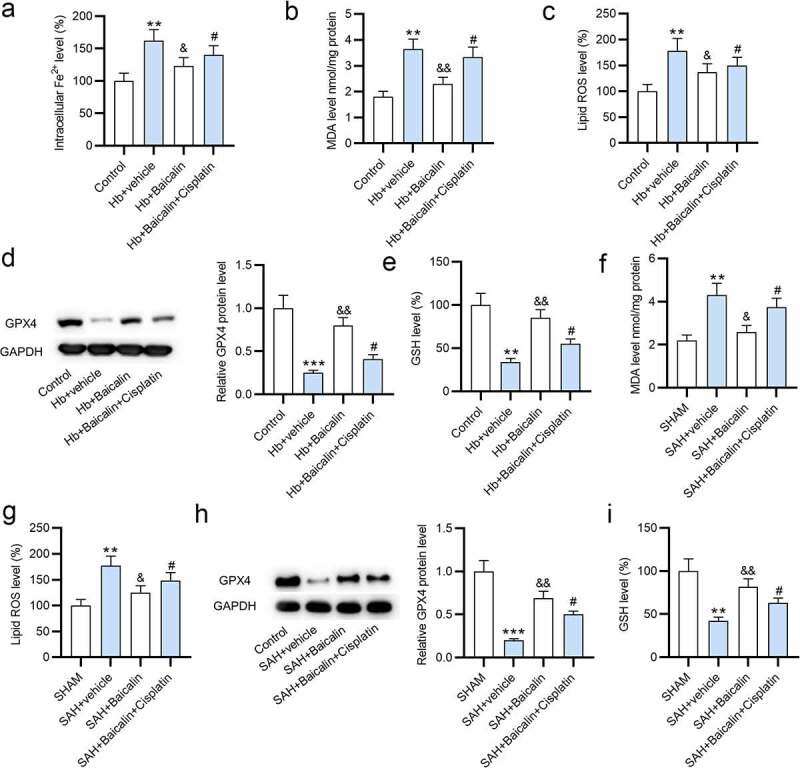
**Effects of baicalin on Fe^2+^, MDA, GSH, and ROS levels**. (a) The levels of Fe^2+^, (b) MDA, and (c) ROS in the primary neurons in 4 groups: (i) Control, (ii) Hb + vehicle, (iii) Hb + Baicalin, (iv) Hb + Baicalin + Cisplatin. (d) The protein level of GPX4 was revealed by western blot analysis. (e) The level of GSH in the four groups. (f-i) The measurement of MDA, ROS, GPX4 and GSH in the brain tissues of rats in 4 groups: (i) SHAM, (ii) SAH + vehicle, (iii) SAH + Baicalin, (iv) SAH + Baicalin + Cisplatin. There are 10 rats in each group. **P < 0.01, ***P < 0.001 *versus* control or sham group. ^&^P < 0.05, ^&&^P < 0.01 *versus* Hb + vehicle group or SAH + vehicle group. ^#^P < 0.05 *versus* Hb + Baicalin group or SAH + Baicalin group

Ferroptosis in the in vitro and in vivo models of SAH was evaluated by detecting Fe^2+^, MDA, ROS, and GSH levels. We observed that the level of iron ion was increased following Hb treatment, and the introduction of baicalin reduced the level of iron, which was then countered by cisplatin (Figure 3(a)). In addition, the levels of MDA and lipid ROS were increased by Hb, which were then reduced by baicalin, while the introduction of cisplatin increased the levels of MDA and lipid ROS (Figure 3(b,c)). Subsequently, the protein level of GPX4 was lower in the Hb + vehicle group, and the treatment of baicalin increased GPX4 protein level, which was then countered by cisplatin treatment (Figure 3(d)). Next, we observed that the level of GSH was decreased by Hb treatment, and baicalin increased the level of GSH, which was then offset by cisplatin (Figure 3(e)). The data above suggested that cisplatin offsets the effect of baicalin on Fe^2+^, MDA, ROS, GPX4 protein, and GSH levels in vitro. For animal exploration, the increase of MDA and ROS levels in SAH + vehicle group was repressed by baicalin treatment, and such effect was reversed by cisplatin administration (Figure 3(f,g)). GPX4 protein and GSH levels were lower in SAH + vehicle group than in the sham group. The promotive effects of baicalin on GPX4 protein and GSH level were reversed by cisplatin treatment (Figure 3(h,i)).

### 3.3. Effects of baicalin on beclin1, LC3-II/I levels in the brain tissues of rats

**Figure 4.**
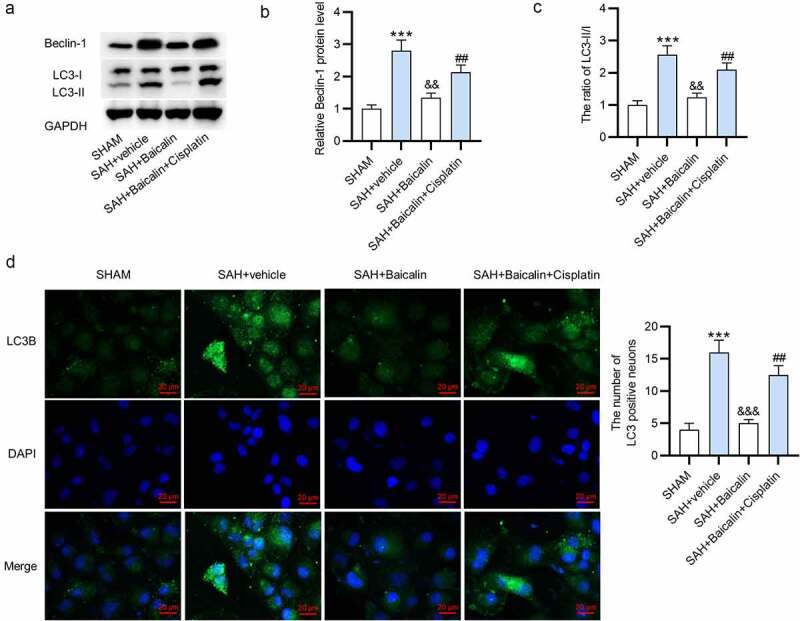
**Effects of baicalin on beclin1, LC3-II/I levels in the brain tissues of rats**. (a) The cortical protein levels of beclin1, LC3-II, and LC3-I after surgery for 24 h. (b) Quantitative data of Beclin-1 protein level 24 h after surgery. (c) The ratio of LC3-II/LC3-I 24 h after surgery. (d) Immunofluorescence staining was conducted to assess LC3 punctuation 24 h after surgery. There are 10 rats in each group. ***P < 0.001 *versus* sham group. ^&&^P < 0.01, ^&&&^P < 0.001 *versus* SAH + vehicle group. ^##^P < 0.01 *versus* SAH + Baicalin group

Effects of baicalin on beclin1, cortical LC3-II/I levels were detected to evaluate autophagy. First, we observed that Beclin-1 protein level and LC3-II/LC3-I ratio were increased after SAH, and baicalin decreased the protein levels of Beclin-1 and ratio of LC3-II/LC3-I, and cisplatin partially abolished the rescue effect of baicalin on SAH-induced autophagy (Figure 4(a,c)). Next, immunofluorescence staining also showed that baicalin treatment decreased LC3 punctuation, and this result was counteracted by cisplatin treatment (Figure 4(d)).

### 3.4. Baicalin suppressed neuronal apoptosis

**Figure 5.**
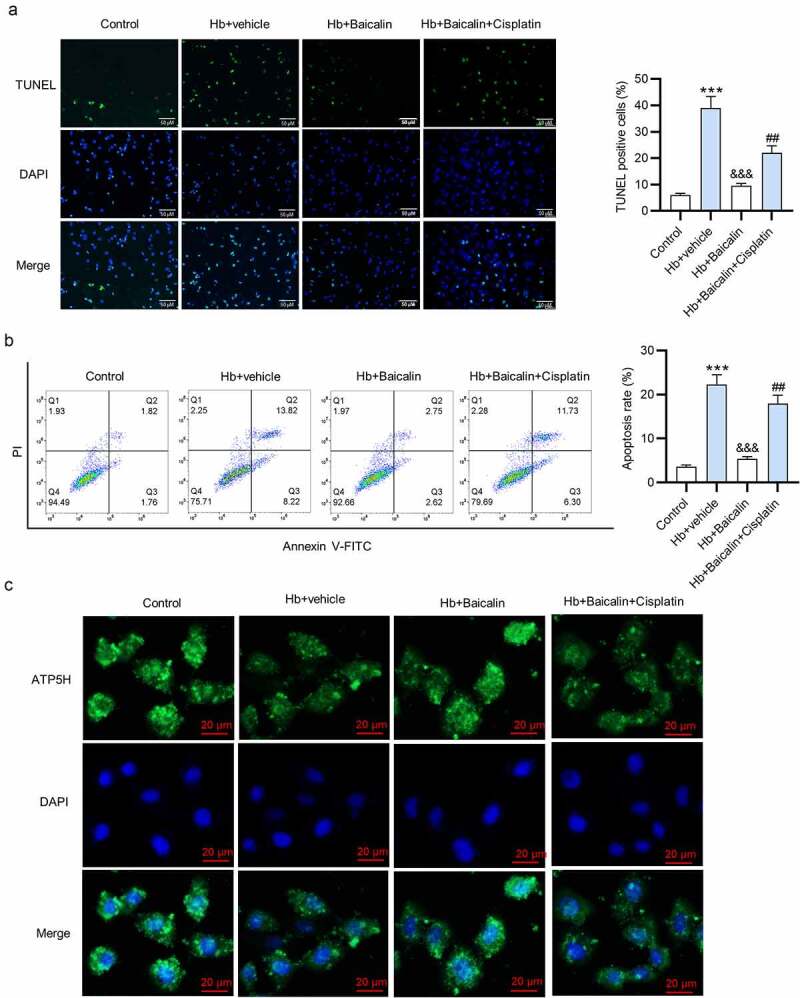
**Effects of baicalin on the cell apoptosis**. (a) Overlapped images showed the TUNEL-positive neurons after different treatments: (i) control; (ii) Hb + vehicle; (iii) Hb + Baicalin; (iv) Hb + Baicalin + cisplatin. (b) Annexin V-FITC/PI staining results were analyzed by flow cytometry analysis to show neuronal apoptosis. (c) Immunofluorescence staining of complex V subunit d to show the mitochondria morphology of the neurons. ***P < 0.001 *versus* control group. ^&&&^P < 0.001 *versus* Hb + vehicle group. ^##^P < 0.01 *versus* Hb + Baicalin group

Subsequently, we evaluated the effects of baicalin on neuronal apoptosis. Figure 5(a) shows that the percentage of TUNEL-positive neurons was higher in the Hb + vehicle group than in the control group, and baicalin reduced the percentage, which was then abrogated by cisplatin treatment. According to results of flow cytometry analysis, neuronal apoptosis rate was decreased by baicalin, and increased by cisplatin (Figure 5(b)). Figure 5(c) revealed that baicalin rescued the Hb-induced mitochondria damage, and the rescue effect of baicalin was suppressed by cisplatin.

### 3.5. Baicalin amoralities brain injury of the SAH rats

**Figure 6.**
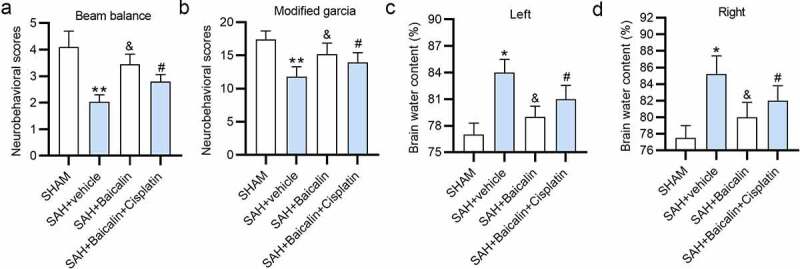
**Effects of baicalin on brain injury of the SAH rats**. (a) The beam balance scores of the rats in the different groups: (i) sham, (ii) SAH + vehicle, (iii) SAH + Baicalin, (iv) SAH + Baicalin + Cisplatin 24 h post-SAH. (b) The modified Garcia scores of the rats 24 h post-SAH. (c and d) The brain water content (%) of the rats 24 h post-SAH. There are 10 rats in each group. *P < 0.05, **P < 0.01 *versus* sham group. ^&^P < 0.05 *versus* SAH + vehicle group. ^#^P < 0.05 *versus* SAH + Baicalin group

Finally, we assessed the influence of baicalin on brain injury of the SAH rats by performing rescue assays. Baicalin increased the beam balance scores and modified Garcia scores of the rats, which were then reduced by cisplatin (Figure 6(a,b)). In addition, baicalin countervailed the SAH-induced increase of brain water content of the left and right cerebral hemispheres, and cisplatin counteracted the rescue effect of baicalin in the brain water content (Figure 6(c,d)).

## 4. Discussion

Previously, research has disclosed that ferroptosis is involved in lots of conditions, such as traumatic brain injury [36,37], ischemia-reperfusion injury [38–40] and intracerebral hemorrhage [41,42]. Particularly, ferroptosis was found to participate in EBI after SAH [17,18]. In the present study, we confirmed the activation of autophagy-dependent ferroptosis in EBI after SAH. In addition, we verified the protective mechanism of baicalin in autophagy-dependent ferroptosis in EBI after SAH.

First, we found that the beam balance scores and modified Garcia scores of the rats after SAH were lower compared to the rats in the sham group. Our data are consistent with the previous findings that the neurobehavioral scores of the mice/rats after SAH are lower in comparison with the sham-operated animals [28,43].

Subsequently, we focused on the role of baicalin in autophagy-dependent ferroptosis in EBI after SAH. Experimental results demonstrated that baicalin attenuated SAH-induced elevation of the levels of Fe^2+^, MDA, and ROS in the brain tissues of rats. We also found that baicalin abolished the SAH-induced decrease of GPX4 protein level and GSH level. GPX4 is a member of the GSH peroxidases with cytosolic, nuclear, and mitochondrial forms [44,45]. GPX4 reduces phospholipid hydroperoxide, and inhibits lipoxygenase-mediated lipid peroxidation, thus exerting a protective effect on ferroptosis [44,46]. Moreover, we observed that baicalin inhibited the protein level of beclin1, the ratio of LC3-II/I and LC3 punctuation in the brain tissues of SAH rats, suggesting the suppressive effect of baicalin on autophagy in SAH rats. Conversely, the inhibitory effect of baicalin was reversed by cisplatin (an inhibitor for both ferroptosis and autophagy). Furthermore, our data manifested that baicalin inhibited the apoptosis and alleviated mitochondria damage to neurons isolated from the rats, and the inhibitive effect was then countervailed by the introduction of cisplatin. Finally, we found that administration of baicalin improved the beam balance scores and modified Garcia scores of the rats after SAH, highlighting the protective role of baicalin against the EBI after SAH.

Previously, baicalin has been already reported to reduce EBI after SAH in rats [28,30]. However, the potential regulatory mechanism of baicalin differs. Previous studies focused on the effects of baicalin on inflammatory response and the impairment of blood-brain barrier by targeting NF-κB signaling. Our study further demonstrated the effects of baicalin on ferroptosis and autophagy via the autophagy signaling pathway.

## 5. Conclusion

Our study revealed that baicalin alleviated EBI after SAH possibly by suppressing autophagy-dependent ferroptosis. Our findings exhibited another regulatory mechanism of baicalin in EBI after SAH, which may deepen our further understanding for the pathology of the EBI after SAH and help develop more effective methods using baicalin for the treatment of this disease.
